# Crosstalk Between mTOR and NF-κB Signaling Pathways in Clear Cell Renal Cell Carcinoma

**DOI:** 10.3390/ijms27177636

**Published:** 2026-08-26

**Authors:** Melanie Glueck, Alexandra Lucaciu, Sumedha Inukollu, Rushendhiran Kesavan, Amelie Janssen, Josef Pfeilschifter, Julien Subburayalu, Ramesh K. Krishnan, Rajkumar Vutukuri

**Affiliations:** 1 Institute of General Pharmacology and Toxicology, Pharmazentrum Frankfurt, Goethe University, 60596 Frankfurt am Main, Germany; m.glueck@blutspende.de (M.G.); lucaciu@med.uni-frankfurt.de (A.L.); ameliejanssen@icloud.com (A.J.); pfeilschifter@em.uni-frankfurt.de (J.P.); 2 Department of Neurology, Goethe University, 60596 Frankfurt am Main, Germany; 3 Department of Bioinformatics, Pondicherry University, Puducherry 605014, India; sumedhas634@gmail.com; 4 Children’s Medical Center Research Institute, University of Texas Southwestern Medical Center, 5323 Harry Hines Boulevard, Dallas, TX 75390, USA; rushendhiran.kesavan@utsouthwestern.edu; 5 Department of Internal Medicine, University Hospital Carl Gustav Carus TU Dresden, Fetscherstraße 74, 01307 Dresden, Germany; julien.subburayalu@tu-dresden.de; 6 Center for Regenerative Therapies Dresden (CRTD), TU Dresden, Fetscherstraße 105, 01307 Dresden, Germany; 7 German Cancer Consortium (DKTK), Partner Site Dresden, and German Cancer Research Center (DKFZ), 69120 Heidelberg, Germany; 8 Department of Medicine, Hematology/Oncology, University Hospital Frankfurt, Goethe University, 60596 Frankfurt am Main, Germany; 9 Frankfurt Cancer Institute, Goethe University, 60596 Frankfurt am Main, Germany

**Keywords:** renal cell carcinoma, mTOR signaling, NF-κB signaling, inflammation, cell proliferation

## Abstract

Clear cell renal cell carcinoma (ccRCC) is the most common and aggressive type of renal cell carcinoma (RCC), representing approximately 80% of cases globally. Despite improved diagnosis and therapy, treatment of aggressive or metastatic ccRCC remains challenging due to acquisition of primary or secondary resistance. Among the dysregulated signaling mechanisms identified in ccRCC, the mechanistic target of rapamycin (mTOR) and the nuclear factor kappa light-chain enhancer of activated B cells (NF-κB) pathways play central roles in regulating various biological functions such as metabolism, inflammation, tumor growth, and survival. However, the molecular crosstalk between mTOR and NF-κB signaling in ccRCC progression and therapeutic resistance remains poorly understood. Therefore, in our current study, we aimed to investigate the interplay between mTOR and NF-κB signaling in ccRCC. We analyzed tumor tissue samples from human ccRCC patients. For validation of mTOR and NF-κB signaling, we used two human ccRCC cell lines, A498 and 786-O. Using pharmacological inhibitors of mTOR and IKK/NF-κB signaling, Torin-1 and MLN120B, respectively, we assessed the functional relationship between these two pathways employing immunoblotting, EdU-based immunocytochemistry, and functional assays. Our findings reveal that both mTOR and NF-κB pathways are aberrantly activated in human ccRCC tissues. Phosphorylation of IκBα, S6, and 4E-BP1 was increased compared with matched adjacent control tissue. In A498 and 786-O cells, pharmacological inhibition of mTOR or IKK/NF-κB altered key readouts of the reciprocal pathway, including AKT, S6, 4E-BP1, IκBα and p65 phosphorylation. Both inhibitors reduced cell number and EdU incorporation, with stronger anti-proliferative effects observed after Torin-1 treatment. Pharmacological inhibition of either pathway altered key readouts of the other pathway, supporting a reciprocal functional association between mTOR- and NF-κB-associated signaling in the ccRCC models analyzed. Our findings support a functional association between mTOR- and NF-κB-associated signaling in the ccRCC models and provide a rationale for further mechanistic studies evaluating combined pathway modulation.

## 1. Introduction

Renal cell carcinoma (RCC) represents a spectrum of malignancies originating from epithelial cells of the renal tubules. It accounts for approximately 2% of global cancer incidence and mortality, with clear cell RCC (ccRCC) being the most prevalent and lethal subtype, representing 80% of all RCC cases [1,2]. While localized tumors are often amenable to surgical resection, approximately 30% of ccRCC patients progress to the metastatic stage. Despite improvements in targeted therapies and immunotherapies [3,4,5], the prognosis for patients with advanced or metastatic ccRCC remains poor, largely due to intrinsic and acquired resistance mechanisms and the highly adaptable nature of the tumors [2,6].

Prior research in ccRCC has identified profound dysregulation of several signaling networks that orchestrate cellular growth, metabolism, immune modulation, inflammation, autophagy, and stress responses [7,8,9,10]. Aberrant activation of survival pathways and evasion of cell death mechanisms enable tumor cells to thrive and resist therapeutic interventions. Accordingly, unraveling the molecular map of perturbed signaling pathways during ccRCC progression is indispensable for identifying novel therapeutic strategies to improve outcomes for patients with ccRCC. Among these, mTOR signaling plays an important role as a master regulator of key cellular processes in RCC [11]. mTOR executes its functions via two distinct complexes, mTORC1 and mTORC2, which influence cellular responses to growth factors, metabolites, and inflammation. These processes are significantly altered in ccRCC [11]. mTORC1 activation enhances mitogen-activated protein kinase 1 (MEK1) expression and triggers extracellular signal-regulated kinase 1 (ERK1) pathway activation, while it simultaneously induces mitogen-activated protein kinase kinase 6 (MKK6)-p38-MAPK signaling and plays a tumor-suppressive role by upregulating p53 and p16. Loss of p38α, p53, or p16 can enhance tumorigenesis, suggesting that evasion of anti-tumoral feedback from the p38-MAPK–p53/p16 axis may contribute to mTOR-driven RCC [11,12]. Accordingly, mTOR inhibitors, such as everolimus and temsirolimus, have been approved for the treatment of advanced RCC. However, their clinical efficacy is often limited due to compensatory activation of alternative pro-survival pathways such as the NF-κB signaling cascade [13]. Selective inhibition of mTORC1 can relieve the negative feedback exerted by the mTORC1/S6K axis on upstream PI3K/AKT signaling, allowing reactivation of AKT, which may subsequently enhance NF-κB signaling through IKK-dependent mechanisms, leading to NF-κB activation, which promotes tumor cell survival, inflammatory signaling, and therapeutic resistance [14].

Parallel to metabolic signaling pathways, inflammatory transcriptional programs are also frequently dysregulated in ccRCC. Here, the NF-κB pathway functions as a master regulator of inflammation, immune response, cell proliferation, and survival. Indeed, its aberrant activation has been reported in various malignancies, including ccRCC [15,16]. Under homeostasis, NF-κB p65 is retained in the cytoplasm by the inhibitor of nuclear factor of kappa B alpha (IκB). In response to various stimuli such as cytokines or oncogenic factors, IκB can be phosphorylated by the IκB kinase (IKK) complex, leading to its subsequent degradation, which allows NF-κB subunits to shuttle into the nucleus [17,18].

Emerging evidence suggests a functional crosstalk between mTOR and NF-κB signaling pathways in several cancers, wherein both pathways can co-regulate cellular processes critical for tumor progression and therapeutic resistance [9,10]. For example, mTOR can modulate NF-κB signaling either directly through interaction with the IκB kinase (IKK) complex components or indirectly via downstream effectors such as S6K1 and AKT, influencing NF-κB nuclear translocation and its transcriptional activity. Conversely, NF-κB activity has been shown to regulate components of the mTOR axis, suggesting the existence of reciprocal regulatory mechanisms [19,20]. However, the precise nature and functional consequence of mTOR–NF-κB crosstalk in the pathogenesis of ccRCC remain poorly defined.

Given the clinical relevance of both pathways in ccRCC and their potential interaction, elucidating the molecular interplay between mTOR and NF-κB signaling could provide critical insights into the development of novel therapeutic strategies to treat patients diagnosed with ccRCC. In this study, we investigated the changes in the mTOR and NF-κB pathways in human ccRCC patient samples and evaluated the functional association of mTOR and NF-κB signaling using the ccRCC cell lines A498 and 786-O. We found that mTOR and NF-κB cascades were upregulated in human ccRCC patient tissues, which may have implications for tumor progression and the response to targeted therapies.

## 2. Results

### 2.1. Enhanced Activation of mTOR and NF-κB Signaling in Human ccRCC Patient Samples

**Figure 1:**
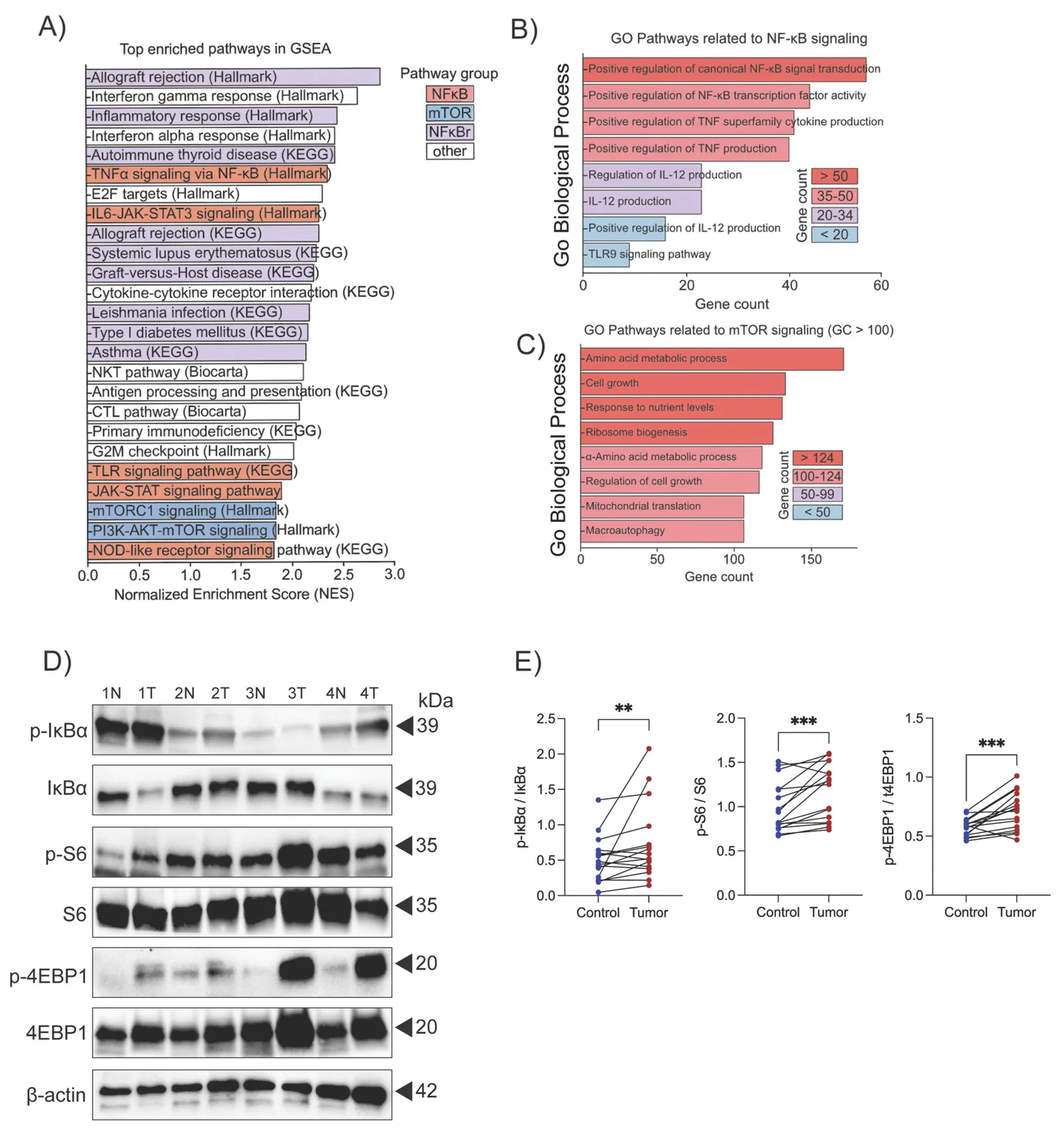
**Enhanced mTOR and NF-κB signaling in human ccRCC patient samples.** (**A**) In silico pathway enrichment analysis from Hu et al. ccRCC cohort [21] showing top regulated pathways in human RCC patient samples; NFκBr: NF- κB-related signaling. (**B**) Altered Gene Ontology (GO) terms associated with NF-κB and related signaling pathways in human RCC patient samples. (**C**) Altered GO pathways belonging to mTOR signaling in human RCC patient samples. (**D**) Western blot analysis of human RCC patient samples. Representative Western blots show four matched patient pairs from a total of 16 matched tumor/adjacent normal tissue pairs analyzed. “T” indicates tumor tissue. “N” indicates normal tissue, which is adjacent to the tumor samples of the same patient, confirmed by histological analysis by a pathologist. (**E**) Densitometric quantification of phosphorylated IκBα (p-IκBα), S6 (p-S6), and 4E-BP1 (p4E-BP1) normalized to their respective total protein levels in matched normal tissue and tumor samples. Each data point represents an individual patient pair, and lines connect matched samples. Tumor tissues showed significantly increased phosphorylation of IκBα, S6, and 4E-BP1 compared with matched normal controls, indicating increased NF-κB-associated activation markers and mTORC1 pathway activity. Statistical analysis was performed using the two-tailed Wilcoxon matched-pairs signed rank test. *p* values: p-IκBα, *p* = 0.0098 (*n* = 16); p-S6, *p* = 0.0020 (*n* = 16); 4E-BP1, *p* = 0.0020 (*n* = 16). ** indicates *p* ≤ 0.01 and *** indicates *p* < 0.001.

To evaluate the clinical relevance of the mTOR and NF-κB pathways in ccRCC, we first performed data mining using an existing human RCC multi-omics dataset of over 100 patients from a recently published article by Hu et al. [21]. Our transcriptomic analysis demonstrated significantly increased expression of multiple genes encoding upstream regulators and downstream effectors of both the NF-κB and mTOR signaling pathways in ccRCC compared with healthy kidney tissues (Figure 1A–C). These included pathways regulating protein synthesis, metabolism, autophagy, and inflammation, which are directly influenced by either mTOR or NF-κB signaling (Figure 1A–C). To further validate these in silico bioinformatic findings at the protein level, we next analyzed matched normal adjacent kidney tissue and tumor tissue from human ccRCC patients by Western blotting. Our results revealed increased phosphorylation of key NF-κB- and mTOR-associated signaling proteins in tumor tissues compared to paired normal tissues (Figure 1D). In line with the pathway enrichment results, phosphorylated IκBα and phosphorylated forms of the mTORC1 downstream targets S6 and 4E-BP1 were also elevated in tumor tissues, indicating enhanced activation of the NF-κB pathway and mTOR-associated translational signaling, respectively.

Densitometric quantification of matched patient samples confirmed a significant increase in p-IκBα, p-S6, and p-4E-BP1 levels in tumor tissues compared with corresponding normal tissues (Figure 1E). Together, these data support the activation of both NF-κB- and mTOR-associated signaling pathways in human ccRCC tissues and provide a clinical basis for further examining their functional crosstalk in RCC.

### 2.2. Pharmacological Inhibition Reveals a Reciprocal Modulation of mTOR and NF-κB-Associated Signaling in A498 and 786-O RCC Cell Lines

**Figure 2:**
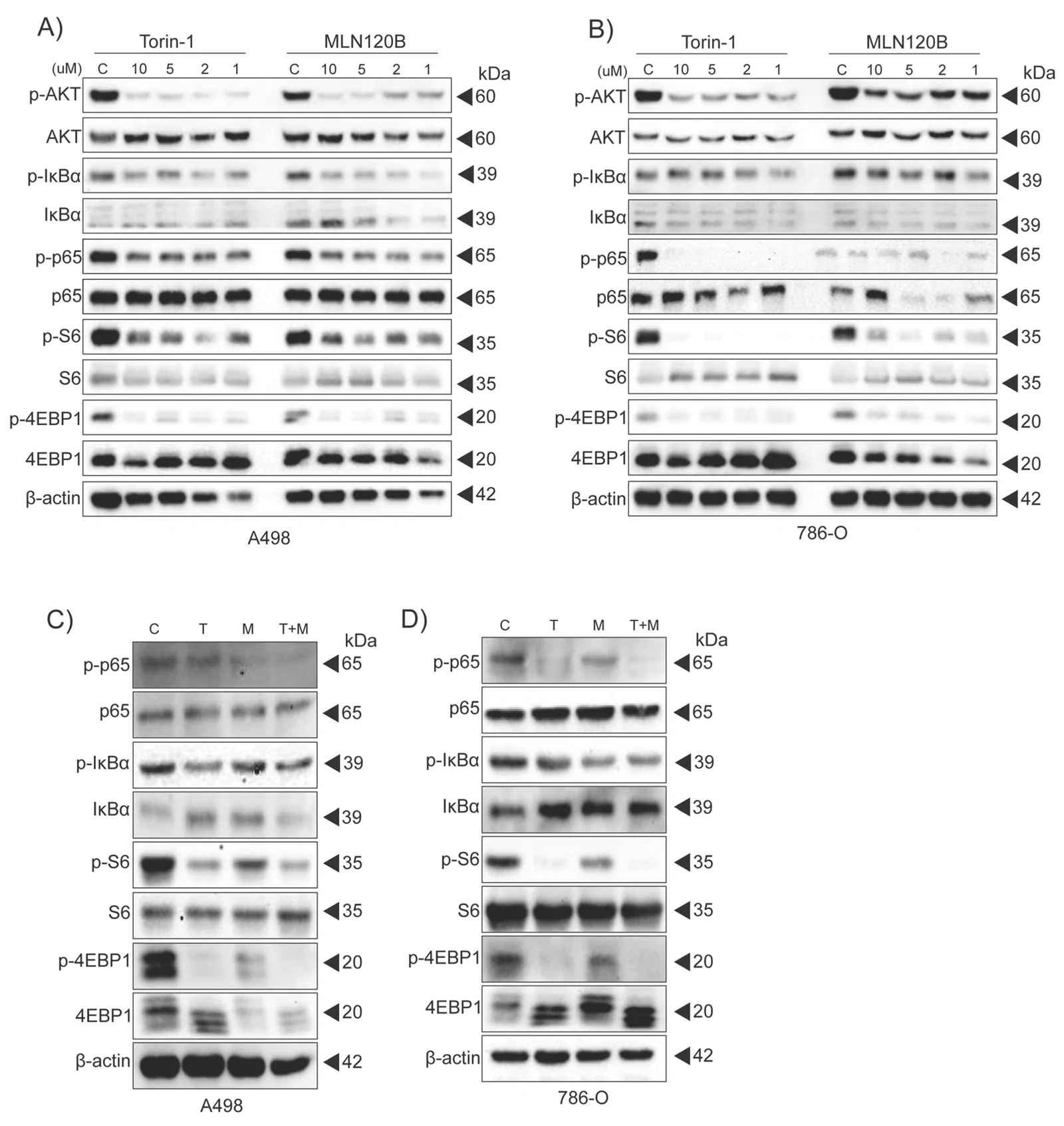
Pharmacological inhibition of NF-κB and mTOR signaling results in their reciprocal suppression in the RCC cell lines A498 and 786-O. (**A**) Western blot analysis of mTORC1/2 inhibition by Torin-1 and NF-κB inhibition using MLN120 in A498 cells. Changes in the expression of different target proteins in both the NF-κB and mTOR signaling pathways are shown. (**B**) Western blot analysis of mTORC1/2 inhibition using Torin-1 and NF-κB inhibition using MLN120B in 786-O RCC cells. Changes in the expression of different target proteins in both the NF-κB and mTOR signaling pathways are shown. Representative blots from *n* = 4 independent experiments are shown. Western blot analysis using (**C**) A498 or (**D**) 786-O cells that were treated with either Torin-1 (10 µM) or MLN120B (10 µM) or together. Changes in the expression of different target proteins in both the NF-κB and mTOR signaling pathways are shown. C-Control, T-Torin, M-MLN120B, T + M (Torin + MLN120B).

To investigate the reciprocal/functional association between the mTOR and NF-κB pathways in ccRCC, we used two common RCC cell lines and treated them with MLN120B, an IKKβ inhibitor that suppresses canonical IKK/NF-κB-associated signaling, or Torin-1, an mTOR inhibitor. At the concentrations used, MLN120B and Torin-1 are widely reported to selectively inhibit IKKβ and mTOR kinase activity, respectively. RCC cells treated with MLN120B showed reduced phosphorylation of IκBα, consistent with inhibition of IKK/NF-κB-associated signaling, and also showed decreased phosphorylation of AKT, S6, and 4E-BP1 (Figure 2A,B). Similarly, inhibition of mTORC1/2 by Torin-1 in ccRCC cells efficiently suppressed the phosphorylation of mTOR downstream targets, including AKT, S6, and 4E-BP1 (Figure 2A,B). Notably, Torin-1 also significantly reduced the phosphorylation of IκBα and NF-κB p65, thereby indicating that mTOR inhibition also attenuates NF-κB signaling in ccRCC. These results indicate that blocking mTOR signaling not only dampens protein synthesis but also reduces NF-κB-associated signaling markers in RCC cells. Together, these results show that pharmacological inhibition of either mTOR- or NF-κB-associated signaling alters the signaling of the other pathway, supporting a reciprocal functional association between these signaling networks in RCC cells. It is important to note that conclusions regarding NF-κB signaling are based on changes in the phosphorylation of IκBα and NF-κB p65, which represent upstream regulatory events rather than a direct measure of NF-κB transcriptional activity. Next, to determine whether simultaneous blockade of both signaling pathways produces further suppression, we treated A498 and 786-O cells with vehicle control, Torin-1, MLN120B, or the combined Torin-1/MLN120B treatment, with both inhibitors used at 10 µM. Western blot analysis was performed for the same NF-κB- and mTOR-associated signaling proteins. As shown in Figure 2A,B, Torin-1 or MLN120B treatment reduced phosphorylation of mTOR and NF-κB-associated pathway effectors. Notably, combined treatment resulted in a greater reduction in both NF-κB- and mTOR-associated signaling proteins in A498 and 786-O cells, indicating that simultaneous pathway inhibition substantially suppresses both signaling networks (Figure 2C,D).

These observations further support a functional dependence between mTOR and NF-κB signaling and indicate that combined pharmacological inhibition produces greater suppression of the pathway proteins analyzed than a single-inhibitor treatment in RCC cells, suggesting that dual pharmacological treatment produces a stronger reduction in tumorigenic pathway activity than single-pathway inhibition alone.

### 2.3. Targeting of NF-κB- and mTOR-Related Signaling Reduces Proliferation of ccRCC Cells

**Figure 3:**
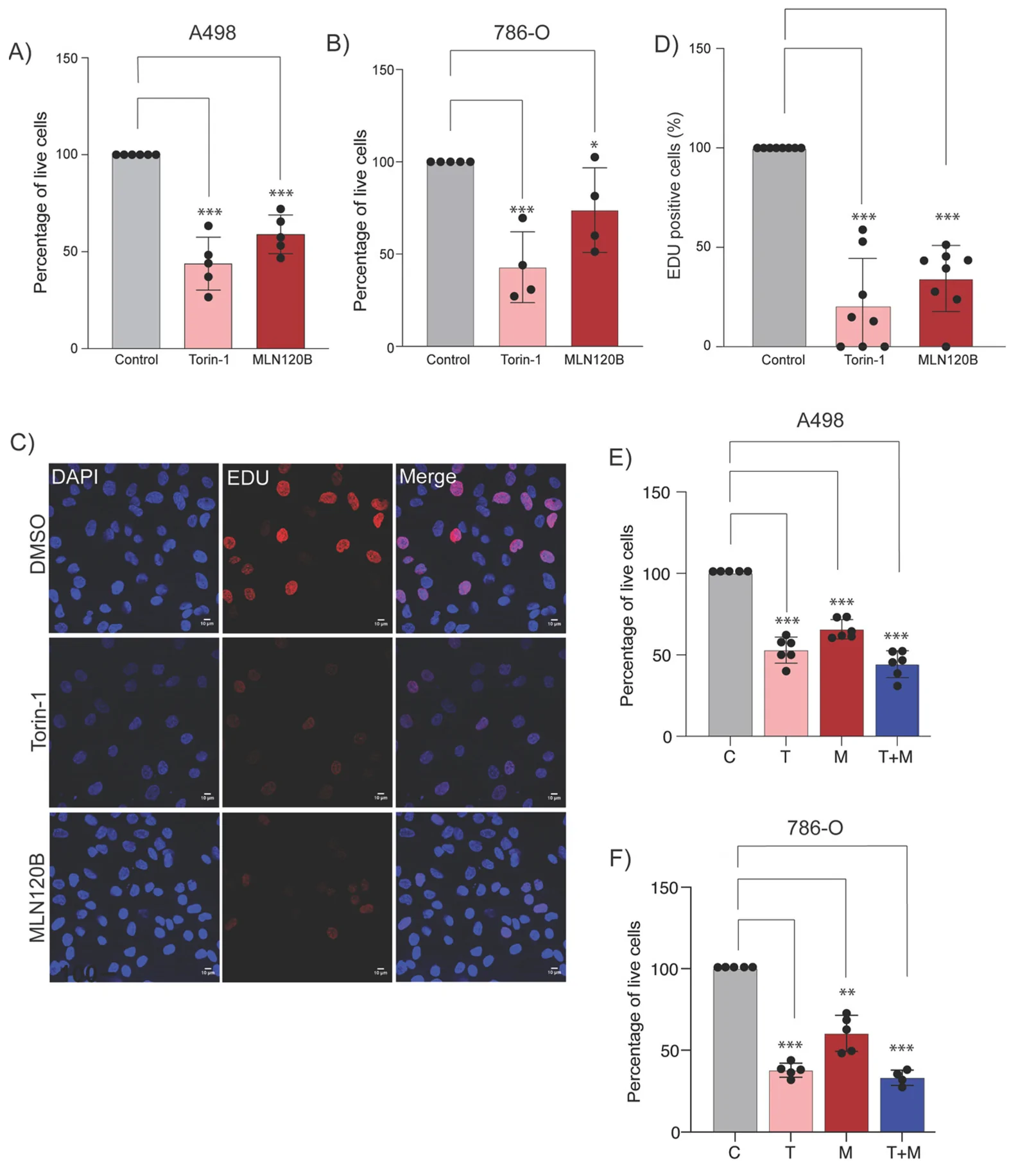
**Pharmacological inhibition of NF-κB- and mTOR-associated signaling reduces live-cell numbers and EdU incorporation in RCC cells.** (**A**) A498 and (**B**) 786-O cells were treated with 0.5 µM Torin-1 and 10 µM MLN120B. After 48 h, viable cells were counted using an automatic cell counter. *n* = 5 individual experiments were performed. (**C**) EdU incorporation assay in A498 cells after 48 h treatment, showing percent EdU-positive cells (*n* = 3 individual experiments). (**D**) Quantification of the number of EdU-positive cells/total number of nuclei using ImageJ 2.16.0/1.54p/java 21.0.7 (64-bit). (**E**,**F**) Quantification of viable A498 and 786-O cell numbers following treatment with vehicle control, Torin-1 (10 µM), MLN120B (10 µM), or combined Torin-1 + MLN120B (10 µM) treatment. Cells were counted after 24 h of treatment using an automated cell-counting approach. * *p* < 0.05, ** *p* < 0.01, *** *p* < 0.001 versus control. C, Control; T, Torin; M, MLN120B; T + M, Torin + MLN120B.

Furthermore, we evaluated the functional consequences of inhibiting the NF-κB and mTOR pathways in both A498 and 786-O cells. For this purpose, we quantified live-cell numbers after 48 h of treatment with pathway inhibitors. In both A498 and 786-O cell lines, both inhibitors significantly reduced live-cell numbers, most prominently after Torin-1 treatment (Figure 3A,B). Indeed, Torin-1, when compared with MLN120B, showed a significantly greater reduction in cell viability (Figure 3A,B), suggesting that, under the experimental conditions used, mTOR inhibition exerted a stronger anti-proliferative effect than IKK/NF-κB inhibition. To address this, we treated cells with the thymidine analog dye 5-ethynyl-2′-deoxyuridine (EdU) for 16 h after treatment with vehicle control (DMSO), Torin-1, or MLN120B. Analysis of the tumor cells after 48 h revealed that treatment with Torin-1 reduced the incorporation of EdU more strongly compared to the inhibition of NF-κB (Figure 3C,D). To further examine the effect of dual inhibition of both signaling pathways on RCC cell viability, we performed viable cell counting for the following treatment conditions: vehicle control, Torin-1, MLN120B, and combined Torin-1/MLN120B treatment. In A498 cells, both single-inhibitor treatments reduced live cell numbers compared with vehicle-treated cells, while the combined treatment further reduced the viable cell count (Figure 3E). A similar response was observed in 786-O cells, where inhibition of mTOR or NF-κB signaling decreased viable cell numbers, and the combined treatment produced a greater reduction than single-pathway inhibition alone (Figure 3F).

Together, these functional assays indicate that pharmacological inhibition of mTOR- and NF-κB-associated signaling reduces RCC live-cell numbers and proliferative activity. These data further support that simultaneous pathway inhibition can produce a broader anti-proliferative effect in both A498 and 786-O cells.

## 3. Discussion

In the present study, we performed data mining of a publicly available human RCC patient dataset and performed mechanistic validation experiments in common ccRCC cell lines to evaluate the relationship between mTOR and NF-κB signaling. Our transcriptomic analysis of the patient cohort confirmed enhanced mTOR-related metabolic pathways and NF-κB-regulated inflammatory networks in human ccRCC [21]. Western blot analyses of human RCC samples supported these bioinformatic findings by showing higher levels of NF-κB- and mTOR-associated pathway markers. Accordingly, inhibition of NF-κB signaling in human RCC cell lines using MLN120B decreased p-IκBα and p-p65 protein levels and, surprisingly, also significantly reduced p-AKT, which is an important activator of mTOR signaling. Likewise, selective inhibition of mTORC1/2-associated signaling with Torin-1 suppressed canonical mTOR pathway proteins (p-S6, p-4E-BP1) and reduced p-AKT, p-IκBα, and p-p65.

Our results, in which both inhibitors decreased p-AKT, could suggest that AKT serves as a central link between the PI3K–AKT–mTOR axis and IKK–IκBα–NF-κB signaling, as previously hypothesized [22]. AKT has been reported to promote activation of the IKK complex in certain contexts, thereby facilitating IκBα phosphorylation and canonical NF-κB pathway activation. In contrast, NF-κB signaling has been reported to regulate PI3K/AKT activity indirectly, promoting a feed-forward loop in some contexts [13,22].

Several explanations can account for the effects of Torin-1 in our proliferation assay. First, mTOR directly controls protein synthesis, metabolic programs, and cell-cycle progression via p70S6K and 4E-BP1, producing rapid cell-intrinsic effects on biosynthesis and proliferation [7,23]. Second, mTOR inhibition may compromise survival signaling through multiple effectors (e.g., suppression of the translation of anti-apoptotic proteins), whereas MLN120B primarily suppresses inflammatory signaling, which can be substituted, especially in the tumor microenvironment (TME). Third, Torin-1 (an ATP-competitive inhibitor) has been reported to block both mTORC1 and mTORC2. mTORC2 is a key upstream activator of AKT Ser473 phosphorylation, and its inhibition can rapidly reduce AKT-associated survival signaling and sensitize cells to apoptosis. This is consistent with reports that inhibitors targeting mTORC2 or both complexes often exert stronger anti-proliferative effects than rapalogs that primarily inhibit mTORC1 [23].

In a clinical context, mTOR inhibitors such as everolimus and temsirolimus have shown promise in treating advanced RCC [8,11,24]. However, these therapies often face limitations due to compensatory activation of alternative survival pathways, including NF-κB signaling. In the present study, Torin-1 was used as an mTOR kinase inhibitor that suppresses mTORC1/2-associated signaling rather than acting as a rapalog-type selective mTORC1 inhibitor. Therefore, the compensatory PI3K/AKT activation described after selective mTORC1 inhibition should be distinguished from the effects observed here. In our RCC cell models, Torin-1 reduced phosphorylation of mTOR downstream targets and was accompanied by reduced NF-κB pathway activation markers, supporting a functional association between mTOR activity and NF-κB signaling under these experimental conditions.

Selective mTORC1 inhibition has been reported to impact upstream feedback suppression and thereby promote compensatory PI3K/AKT and NF-κB signaling in some conditions. However, this feedback mechanism differs from the broader mTORC1/2 inhibition produced by Torin-1 under the experimental conditions used in the present study. NF-κB remains a major regulator of inflammation, immune response, and cell survival [22,25,26]. This NF-κB activation can contribute to tumor cell resistance and promote tumor cell survival under stress conditions [27]. Similarly, NF-κB inhibitors have emerged as potential cancer therapies. Several FDA-approved agents used in oncology and inflammatory diseases have been shown to indirectly influence NF-κB signaling [28,29], including proteasome inhibitors like bortezomib, which disrupt NF-κB signaling by preventing the degradation of IκB proteins, thus blocking NF-κB activation. Other NF-κB targeting agents include anti-inflammatory drugs such as corticosteroids and monoclonal antibodies such as bevacizumab, which targets VEGF, or denosumab, which inhibits RANKL; these agents do not directly inhibit NF-κB signaling. However, since NF-κB regulates the expression of VEGF, these agents may indirectly impact NF-κB-driven processes, including angiogenesis and tumor progression, through modulation of downstream pathways and the tumor microenvironment [9,10]. These inhibitors have been approved for various malignancies, including multiple myeloma and solid tumors. However, like mTOR inhibitors, the clinical efficacy of NF-κB inhibitors can be limited by compensatory signaling mechanisms, highlighting the complexity of targeting these pathways individually [7,9,10]. Combined modulation of mTOR- and NF-κB-associated signaling may therefore warrant further investigation as a strategy to suppress complementary proliferative and survival-associated pathway outputs in ccRCC models. This approach could provide a promising therapeutic strategy for ccRCC, potentially enhancing treatment efficacy by addressing both the metabolic and inflammatory survival signals within the TME.

**Figure 4:**
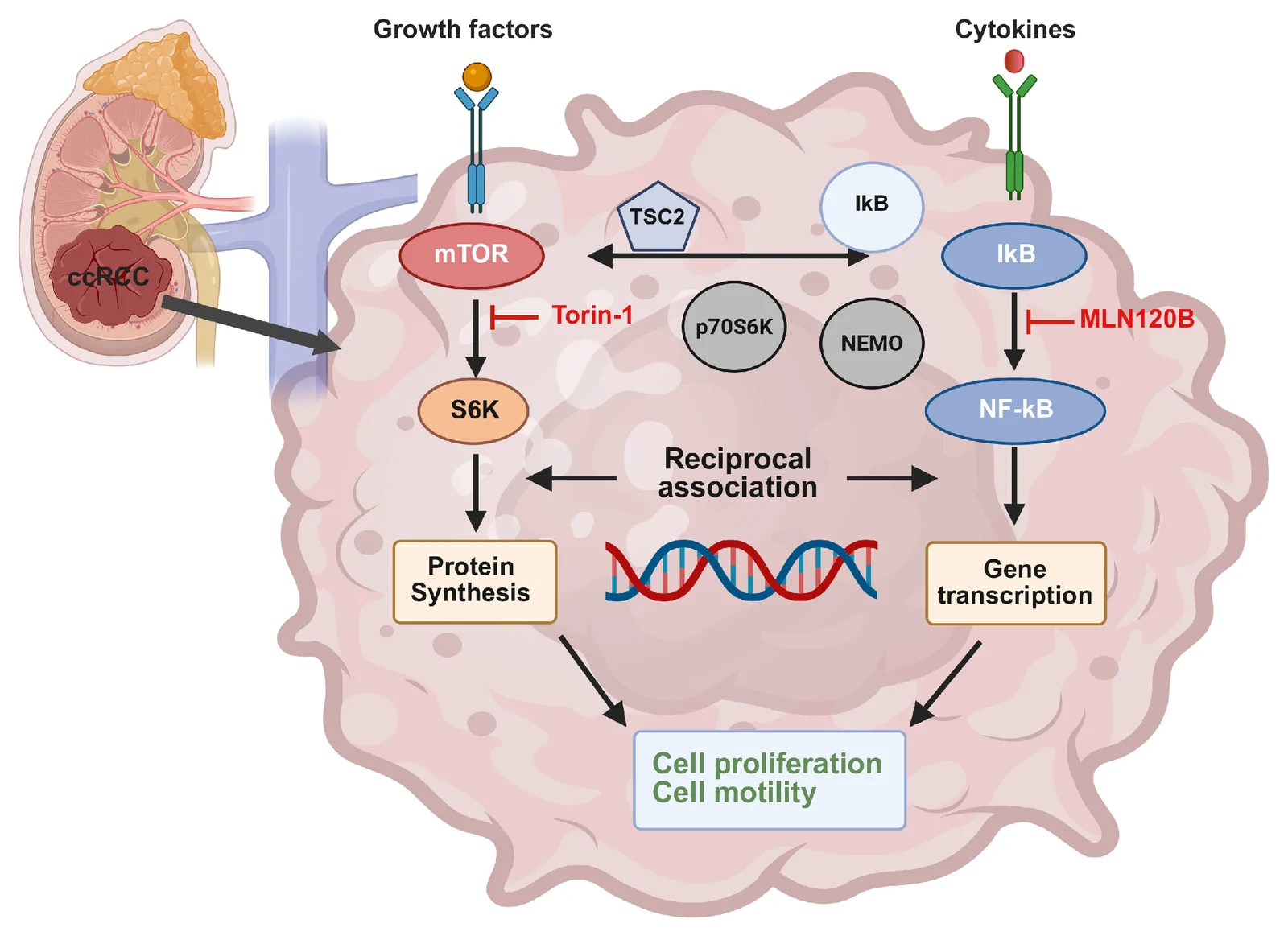
Schematic representation of the functional association between mTOR and NF-κB signaling pathways in ccRCC.

Our results suggest that in ccRCC models in which mTOR inhibition reduces p-AKT and p-IκBα, mTOR inhibitors may have direct anti-proliferative effects and may reduce survival-associated signaling. Whether these effects involve apoptosis requires further investigation using dedicated apoptosis assays. However, because NF-κB signaling supports tumor inflammation, immune modulation, and survival, concurrent strategies that target NF-κB outputs or downstream effectors (for example, specific anti-apoptotic proteins) may further enhance efficacy [30]. Recent reviews have highlighted the promise and challenge of targeting NF-κB in cancer: direct NF-κB blockade can be toxic and is practically complex, but pathway modulators and carefully timed combinations (including metabolic or immunotherapy pairing) are being explored [14,31]. Given the metabolic reprogramming in ccRCC revealed by Hu et al. [21], combining metabolic interventions with mTOR pathway inhibition might be a therapeutic option to explore. Taken together, these findings support a reciprocal modulation of mTOR- and NF-κB-associated pathway readouts in ccRCC models, as summarized in the schematic model (Figure 4).

Schematic illustration depicting the interaction between mTOR and NF-κB signaling pathways in ccRCC. Growth factor–mediated activation of receptor tyrosine kinases stimulates the PI3K–AKT–mTOR axis, leading to activation of mTORC1 and downstream effectors such as S6K, thereby promoting protein synthesis, cell proliferation, and survival. In parallel, cytokine signaling activates the IκB kinase (IKK) complex, resulting in phosphorylation and degradation of IκBα and subsequent activation and nuclear translocation of NF-κB, which drives transcription of genes involved in inflammation, survival, and tumor progression. Crosstalk between the two pathways occurs via AKT-dependent regulation of IKK/NF-κB signaling and reciprocal modulation of mTOR activity by NF-κB-associated mechanisms. Pharmacological inhibition using Torin-1 suppresses mTOR signaling and attenuates NF-κB activity, whereas inhibition of NF-κB using MLN120B reduces IκBα phosphorylation and concurrently dampens mTOR pathway activation. The study supports reciprocal modulation of mTOR- and NF-κB-associated pathways, and this bidirectional interaction contributes to tumor cell proliferation, survival, and therapeutic resistance in ccRCC.

### Limitations

First, the in vitro validation was performed in two established RCC cell lines, A498 and 786-O. Although these models are widely used for studying RCC/ccRCC-associated signaling, they do not capture the full inter-patient and intra-tumoral heterogeneity of ccRCC. Second, pathway interaction was assessed mainly using pharmacological inhibition, which supports a functional association between mTOR and NF-κB signaling but does not establish a direct molecular interaction or a universal causal mechanism. A further limitation is that A498 and 786-O cells, although widely used as RCC/ccRCC-relevant models, differ in origin, phenotype, differentiation state, and molecular background. These differences may contribute to variation in their response to Torin-1 and MLN120B. Therefore, the inhibitor response data should be interpreted as validation in two established RCC cell models rather than as a complete representation of the biological heterogeneity of human ccRCC. Finally, our readout for NF-κB pathway activity is based only on IκBα and NF-κB p65 phosphorylation and does not include NF-κB p65 nuclear translocation or other detailed analyses. Future studies incorporating these analyses will further clarify the mechanistic interplay between these pathways. Moreover, future studies using additional ccRCC cell lines, patient-derived tumor models, organoids, in vivo models, and orthogonal genetic or rescue-based approaches will be required to define the broader relevance and precise mechanistic hierarchy of this pathway relationship.

## 4. Materials and Methods

MLN120B (Catalog No. HY-15473) and Torin-1 (Catalog No. HY-13003) were obtained from MedChemExpress (Monmouth Junction, NJ, USA). All media and cell culture reagents were purchased from Gibco, Thermo Fisher Scientific (Darmstadt, Germany): Dulbecco’s Modified Eagle Medium (DMEM; high glucose, Catalog No. 61965-026), RPMI medium 1640 + Glutamax (Catalog No. 61870-010), Dulbecco’s phosphate-buffered saline (14190144), and penicillin/streptomycin (5000 U/mL). Fetal calf serum (FCS) was obtained from Biochrom KG, Berlin, Germany.

### 4.1. Bioinformatics Analysis

Datasets published by Hu et al. 2024 [21] were extracted, and differential expression analysis was performed using the Limma package version 3.64.3, to obtain a ranked list of genes. The ranked gene list was used for gene set enrichment analysis (GSEA) of KEGG, Biocarta, and Hallmark gene sets using the msigdbr package 25.1.0. A post hoc analysis dot plot was obtained for the top 25 pathways. The code is available upon request from the corresponding author.

### 4.2. Cell Culture

A498 (ATCC HTB-44) and 786-O (ATCC CRL-1932) cells were used as established human ccRCC-relevant cell models. A498 cells are epithelial kidney carcinoma cells originally isolated from the kidney tissue of a 52-year-old female patient with kidney cancer. 786-O cells are adherent renal carcinoma cells derived from a primary clear cell adenocarcinoma of the kidney from a 58-year-old male patient. The 786-O cell line is reported to be tumorigenic in immunosuppressed hamsters and capable of growth in soft agar. Exact clinical tumor-grade information was not consistently available from the original cell-line source information; therefore, we did not assign unsupported Fuhrman/WHO grades. Cell lines were obtained from ATCC (Manassas, VA, USA) and kept between passage numbers 10 and 20. Cell lines were cultured in a humidified 5% CO_2_ environment at 37 °C. For all in vitro experiments in the present study, A498 and 786-O human ccRCC cells were used. A498 cells were maintained in DMEM, and 786-O cells were cultured in RPMI 1640 medium containing 10% (*v*/*v*) FCS and 1% (*v*/*v*) penicillin/streptomycin until confluence. Prior to stimulation, the medium was completely removed and replaced with fresh medium. Cells were treated with different concentrations and time points, as indicated. All chemicals were dissolved according to the manufacturers’ instructions.

### 4.3. Preparation of Whole Protein Lysates

For whole protein lysate preparation from A498 cells following stimulation, the medium was completely aspirated, cells were washed once with PBS and 75 µL of cold lysis buffer (Triton X-100-lysis buffer (1% (*v*/*v*) Triton X-100, 20 mM Tris/HCl, pH 8.0, 137 mM NaCl, 10% (*v*/*v*) glycerol, 1 mM dithiothreitol, 5 mM EDTA, 10 mM sodium fluoride, 2 mM sodium orthovanadate, 1 mM phenylmethylsulfonyl fluoride, 5 ng/mL aprotinin, 5 ng/mL leupeptin, and 50 nM okadaic acid) was added to the cells, which were then scraped and homogenized for 5 min in an ultrasonic bath. For human kidney samples, 100 µL of protein lysis buffer was used for homogenization in an ultrasonic bath for 10 min. The human kidney tissue and cell culture lysates were centrifuged for 15 min at 13,000 rpm; the supernatant was collected, and using the bicinchoninic acid protein assay kit (Pierce 23225; Thermo Fisher Scientific, Darmstadt, Germany), the protein concentration was determined.

### 4.4. Western Blot Analysis

Twenty-five micrograms of protein lysates were analyzed by SDS–polyacrylamide gel electrophoresis (PAGE), transferred onto a 0.2 μm PVDF (polyvinylidene fluoride) (Sigma–Aldrich, Taufkirchen; Germany, Catalog No 03010040001) membrane, blocked with 2.5% (*w*/*v*) non-fat milk, and incubated with primary antibodies overnight at 4 °C. A secondary antibody conjugated to horseradish peroxidase and directed against the respective primary antibody was used. Signals were detected using luminol enhancer solution 32106 (Pierce; Thermo Fisher Scientific, Darmstadt, Germany). The antibodies used in this study include: pNFκB p 65 (Cell signaling technology (CST) 3033, Cell Signaling Technology, Danvers, MA, USA), NF-κB p65 (CST) 8242), pAKT (CST 9271), AKT (CST 9272), pERK (CST 9101), ERK (9102), p4E-BP1 (CST 13443) 4EBP1 (CST 9644), pIκBα (CST 9246), IκBα (CST 9242), CTGF (sc365970, E-5 clone1220), pS6 ribosomal protein (CST 4858), S6 Ribosomal protein (CST 2217) and β-actin antibody (A-2228; Sigma-Aldrich, Taufkirchen, Germany). The dilution of all the primary antibodies used was 1:1000 except for ACTB (1:25,000), and the dilution of all the secondary antibodies was 1:10,000.

### 4.5. Immunocytochemistry

For immunocytochemistry, A498 cells or 786-O cells at a density of 7 × 10^4^ were plated in 8-well chamber, removable plates (Ibidi, 80841, Ibidi, Graefelfing, Germany). Cells were maintained in a humidified 5% CO_2_ environment at 37 °C. The next day, fresh complete medium was added, and cells were treated with MLN120B (10 µM) or Torin-1 (0.5 µM) for 48 h. Following stimulation, cells were treated with 10 µM EdU (C10340, Thermo Fisher Scientific, Waltham, MA, USA) for 16 h, followed by fixation using 1% formalin solution for 10 min at room temperature. The cells were then stained according to the manufacturer’s protocol. Images were acquired using a Leica SP5 confocal microscope (Leica Microsystems CMS GmbH, Wetzlar, Germany). All images, analyses, and quantification were performed in a double-blind fashion to avoid analysis bias.

### 4.6. Statistical Analysis

All data were confirmed in at least three independent experiments. Normally distributed data are shown as mean ± SD, and non-normally distributed data are shown as median ± IQR unless indicated otherwise. For paired comparisons of matched tumor and adjacent normal tissue samples, the two-tailed Wilcoxon matched-pairs signed-rank test was used. To compare more than two conditions involving unpaired samples, one-way ANOVA with Dunnett’s post hoc *t* test for normally distributed data or the Kruskal–Wallis test with Dunn’s post hoc *t* test was used, respectively. Statistical analyses were performed using GRAPHPAD PRISM software version 11.0.2 (GraphPad Software Inc., San Diego, CA, USA). A *p* value < 0.05 was considered statistically significant.

## 5. Conclusions

Our study shows increased mTOR- and NF-κB-associated pathway readouts in human ccRCC tissue samples. Pharmacological inhibition of mTOR or IKK/NF-κB signaling reduced phosphorylation of selected readouts from both pathways and decreased live-cell numbers in A498 and 786-O. Together, our findings indicate that mTOR and NF-κB signaling are functionally associated in human ccRCC tissues and in the RCC cell models analyzed. These data support further investigation of combined pathway modulation, while additional model systems and orthogonal validation will be required before broader therapeutic conclusions can be drawn.

## Data Availability

The original contributions presented in this study are included in the article. Further inquiries can be directed to the corresponding author.

## Abbreviations

The following abbreviations are used in this manuscript:

AKT	Protein kinase B
ccRCC	Clear cell renal cell carcinoma
EdU	5-ethynyl-2′-deoxyuridine
ERK	Extracellular signal-regulated kinase
GO	Gene ontology
IKK	IκB kinase
IκBα	Inhibitor of κB alpha
MAPK	Mitogen-activated protein kinase
mTOR	Mechanistic target of rapamycin
mTORC1	mTOR complex 1
mTORC2	mTOR complex 2
NF-κB	Nuclear factor kappa light-chain enhancer of activated B cells
PI3K	Phosphatidylinositol 3-kinase
p-S6	Phosphorylated ribosomal protein S6
p-4E-BP1	Phosphorylated eukaryotic translation initiation factor 4E-binding protein 1

## References

[1] Young M., Jackson-Spence F., Beltran L., Day E., Suarez C., Bex A., Powles T., Szabados B. (2024). Renal cell carcinoma. Lancet.

[2] Sun J., Chen F., Wu G. (2023). Role of NF-κB pathway in kidney renal clear cell carcinoma and its potential therapeutic implications. Aging.

[3] Powles T., Albiges L., Bex A., Comperat E., Grünwald V., Kanesvaran R., Kitamura H., McKay R., Porta C., Procopio G. (2024). Renal cell carcinoma: ESMO Clinical Practice Guideline for diagnosis, treatment and follow-up. Ann. Oncol..

[4] Motzer R.J., Jonasch E., Agarwal N., Alva A., Bagshaw H., Baine M., Beckermann K., Carlo M.I., Choueiri T.K., Costello B.A. (2024). NCCN Guidelines^®^ Insights: Kidney Cancer, Version 2.2024. J. Natl. Compr. Canc Netw..

[5] Bex A., Ghanem Y.A., Albiges L., Bonn S., Campi R., Capitanio U., Dabestani S., Hora M., Klatte T., Kuusk T. (2025). European Association of Urology Guidelines on Renal Cell Carcinoma: The 2025 Update. Eur. Urol..

[6] Miricescu D., Balan D., Tulin A., Stiru O., Vacaroiu I., Mihai D., Popa C.C., Papacocea R.I., Enyedi M., Sorin N.A. (2021). PI3K/AKT/mTOR signalling pathway involvement in renal cell carcinoma pathogenesis (Review). Exp. Ther. Med..

[7] Jiang A., Li J., He Z., Liu Y., Qiao K., Fang Y., Qu L., Luo P., Lin A., Wang L. (2024). Renal cancer: Signaling pathways and advances in targeted therapies. MedComm.

[8] Hossain M.T., Hossain M.A. (2025). Targeting mTOR Kinase for Cancer Treatment: A Comprehensive Review with Clinical Insights. Drug Dev. Res..

[9] Hideshima T., Ikeda H., Chauhan D., Okawa Y., Raje N., Podar K., Mitsiades C., Munshi N.C., Richardson P.G., Carrasco R.D. (2009). Bortezomib induces canonical nuclear factor-kappaB activation in multiple myeloma cells. Blood.

[10] Sung M.H., Bagain L., Chen Z., Karpova T., Yang X., Silvin C., Voss T.C., McNally J.G., Van Waes C., Hager G.L. (2008). Dynamic effect of bortezomib on nuclear factor-kappaB activity and gene expression in tumor cells. Mol. Pharmacol..

[11] Hudes G., Carducci M., Tomczak P., Dutcher J., Figlin R., Kapoor A., Staroslawska E., Sosman J., McDermott D., Bodrogi I. (2007). Temsirolimus, Interferon Alfa, or Both for Advanced Renal-Cell Carcinoma. N. Engl. J. Med..

[12] Battelli C., Cho D.C. (2011). mTOR inhibitors in renal cell carcinoma. Therapy.

[13] Morais C., Gobe G., Johnson D.W., Healy H. (2011). The emerging role of nuclear factor kappa B in renal cell carcinoma. Int. J. Biochem. Cell Biol..

[14] Jayab N.A., Abed A., Talaat I.M., Hamoudi R. (2025). The molecular mechanism of NF-κB dysregulation across different subtypes of renal cell carcinoma. J. Adv. Res..

[15] Hamieh L., Choueiri T.K., Ogórek B., Khabibullin D., Rosebrock D., Livitz D., Fay A., Pignon J.-C., McDermott D.F., Agarwal N. (2018). Mechanisms of acquired resistance to rapalogs in metastatic renal cell carcinoma. PLoS Genet..

[16] Guo Q., Jin Y., Chen X., Ye X., Shen X., Lin M., Zeng C., Zhou T., Zhang J. (2024). NF-κB in biology and targeted therapy: New insights and translational implications. Signal Transduct. Target. Ther..

[17] Barnes P.J., Karin M. (1997). Nuclear Factor-κB—A Pivotal Transcription Factor in Chronic Inflammatory Diseases. N. Engl. J. Med..

[18] Napetschnig J., Wu H. (2013). Molecular basis of NF-κB signaling. Annu. Rev. Biophys..

[19] O’Reilly K.E., Rojo F., She Q.B., Solit D., Mills G.B., Smith D., Lane H., Hofmann F., Hicklin D.J., Ludwig D.L. (2006). mTOR Inhibition Induces Upstream Receptor Tyrosine Kinase Signaling and Activates Akt. Cancer Res..

[20] Li Z., Su P., Yu M., Zhang X., Xu Y., Jia T., Yang P., Zhang C., Sun Y., Li X. (2024). YAP represses the TEAD-NF-κB complex and inhibits the growth of clear cell renal cell carcinoma. Sci. Signal..

[21] Hu J., Wang S.G., Hou Y., Chen Z., Liu L., Li R., Li N., Zhou L., Yang Y., Wang L. (2024). Multi-omic profiling of clear cell renal cell carcinoma identifies metabolic reprogramming associated with disease progression. Nat. Genet..

[22] Dan H.C., Cooper M.J., Cogswell P.C., Duncan J.A., Ting J.P.Y., Baldwin A.S. (2008). Akt-dependent regulation of NF-{kappa}B is controlled by mTOR and Raptor in association with IKK. Genes. Dev..

[23] Glaviano A., Foo A.S.C., Lam H.Y., Yap K.C.H., Jacot W., Jones R.H., Eng H., Nair M.G., Makvandi P., Geoerger B. (2023). PI3K/AKT/mTOR signaling transduction pathway and targeted therapies in cancer. Mol. Cancer.

[24] Hudes G.R. (2009). Targeting mTOR in renal cell carcinoma. Cancer.

[25] Ghosh S., Tergaonkar V., Rothlin C.V., Correa R.G., Bottero V., Bist P., Verma I.M., Hunter T. (2006). Essential role of tuberous sclerosis genes TSC1 and TSC2 in NF-kappaB activation and cell survival. Cancer Cell.

[26] Kezic A., Becker J.U., Thaiss F. (2013). The effect of mTOR-inhibition on NF-κB activity in kidney ischemia-reperfusion injury in mice. Transplant. Proc..

[27] Mao H., Zhao X., Sun S.C. (2025). NF-κB in inflammation and cancer. Cell. Mol. Immunol..

[28] Miller S.C., Huang R., Sakamuru S., Shukla S.J., Attene-Ramos M.S., Shinn P., Van Leer D., Leister W., Austin C.P., Xia M. (2010). Identification of known drugs that act as inhibitors of NF-kappaB signaling and their mechanism of action. Biochem. Pharmacol..

[29] Olivier S., Robe P., Bours V. (2006). Can NF-kappaB be a target for novel and efficient anti-cancer agents?. Biochem. Pharmacol..

[30] Lukas K., Nguyen J., Necas C., Dave K., Venketaraman V. (2025). Targeting the NF-κB Pathway in Cancer: Mechanisms, Resistance, and Therapeutic Potential Across Tumor Types. Pharmaceuticals.

[31] Li Z., Yang Z., Passaniti A., Lapidus R.G., Liu X., Cullen K.J., Dan H.C. (2016). A positive feedback loop involving EGFR/Akt/mTORC1 and IKK/NF-kB regulates head and neck squamous cell carcinoma proliferation. Oncotarget.

